# The New Status Quo: Enhancing Access to Human–Animal Interactions to Alleviate Social Isolation & Loneliness in the Time of COVID-19

**DOI:** 10.3390/ani11102769

**Published:** 2021-09-23

**Authors:** Zenithson Ng, Taylor Chastain Griffin, Lindsey Braun

**Affiliations:** 1 College of Veterinary Medicine, University of Tennessee, Knoxville, TN 37996, USA; zng@utk.edu; 2 Pet Partners, Bellevue, WA 34894, USA; 3 Human Animal Bond Research Institute (HABRI), Washington, DC 20005, USA; lbraun@habri.org

**Keywords:** human–animal interaction, human–animal bond, animal-assisted interventions, companion animals, service animals, therapy animals, animal-related engagement, pet ownership

## Abstract

**Simple Summary:**

The human–animal bond is a powerful agent for reducing feelings of loneliness and social isolation; however, due to the COVID-19 pandemic, our typical avenues for accessing human–animal interaction (HAI) have been drastically impacted. Because the way we interact with animals has shifted during these times of collective struggle, we have ultimately grown in our appreciation of the human–animal bond. This paper will outline the impact of the pandemic on our relationships with animals, while also discussing the ways in which organizations that facilitate HAI have responded to the pandemic. Preparations for post-pandemic re-entry into the community with our animals will be outlined, and suggestions for future research and best practices on this topic will be provided.

**Abstract:**

Access to human–animal interactions (HAI) have been influenced by the COVID-19 pandemic. Service animals that were trained and accustomed to daily access to public places had to adjust to staying at home. Therapy animals and their handlers who previously visited with many of the populations most vulnerable to the virus have had to halt their programming. Professionals who utilize animal-assisted interventions (AAI) have had to develop new strategies for providing goal-oriented care. Even the landscape for companion animals has been significantly altered, leading to behavioral changes and new practices for pet owners and veterinarians. While animals and their human companions face new challenges, our recognition of the power of the human–animal bond (HAB) has grown, as it provides a vital need for connection during this time of isolation. In this paper, we will not only focus on describing the new status quo related to various kinds of animals and the public’s access to HAI, but will also offer suggestions for sharing the human–animal bond during a time in which physical connections are limited. Organizational insights from the service and therapy animal fields will be explored, and findings related to the auspiciousness of new initiatives, such as animal-related engagement (ARE), will be presented. Recommendations for people who share their lives with any of these kinds of animals will be made to ensure both human and animal welfare. Finally, future research and best practices will be suggested, so we can empirically understand and develop these revised offerings to ultimately bring HAI to a wider audience than ever before.

## 1. Introduction

Research suggests that relationships with pets and companion animals (terms that will herein be used interchangeably) are beneficial to humans when investigated across a wide array of outcome variables. Pet ownership has been correlated with improved physical health and increased social functioning [1,2,3,4,5,6]. Service animals assist in performing a specific task for their handler, and they can also positively influence their handlers’ sense of societal connection [7,8,9,10]. Many studies have been conducted to shed light on the influence of therapy animals on our mental health and social functioning, providing evidence that these animals can help to bolster our psychological functioning, increase our perceptions of social support, and reduce feelings of isolation and loneliness [11,12,13,14,15,16]. However, each of these avenues for connecting with animals faced new challenges in response to the COVID-19 pandemic. The social isolation that resulted from the pandemic yielded a newfound perspective on the relationships we have with companion and assistance animals.

In experiencing the call to alter many of our day-to-day activities, the lives of companion animals also changed. Throughout the pandemic, academic research publications and commissioned market survey research, such as the American Pet Products Association (APPA) Pulse Surveys, have noted an elevation in our collective recognition of the human–animal bond (HAB) as a powerful and protective agent in coping with the challenges associated with trying times [17]. In fact, reported rates of animal adoption sky-rocketed shortly after social distancing orders were put in place [18,19,20]. Pet owners also reported higher rates of connection with their animals and stated they were more prone to notice the needs and state of wellness of their companion animals during this time of isolation [17,21]. In addition, service animals experienced a vital change in lifestyle and shift in purpose for people who relied on them to function in the public. Even in the world of animal-assisted interventions (AAI), where therapy animals were largely prevented from being able to visit with the populations in which they inspire wellness, new ways of interacting within safe parameters were realized.

The purpose of this commentary is to describe the impact the COVID-19 pandemic has had on people with companion animals in the home, on companion animals (including owned pets, therapy animals and service animals) and on human–animal interactions as informed by both existing research and the experiences of leaders within the service animal, therapy animal, and companion animal care industries. Although pets of all species are considered in the discussion, the primary focus will be on dogs and cats. We begin by noting the ways in which the COVID-19 pandemic has elevated the importance of the human–animal bond in people’s lives, and follow with a discussion of the pandemic’s impact on the way we care for animals and access the human–animal bond, from pet spending, care and behavior to the training of service animals, to the manner in which human–animal interactions are provided and accessed. This paper will emphasize the importance of understanding these impacts to inform re-entry considerations that are inclusive of our animals’ health and welfare, and provide suggestions for this process in the “new status quo”. We close by considering the ways in which we can continue to support and investigate HAI within the ever-changing world that we share with our animals.

## 2. Elevated Appreciation for the Human–Animal Bond during Isolation

Though access to animals was impacted across various key domains within the HAI realm because of the pandemic, the relationship we share with our animals has been appreciated at new levels in the midst of the COVID-19 crisis. Throughout multiple research projects conducted mid-pandemic, pet owners reported significant increases in their tendency to turn to their animals for emotional support [17,18,20,21,22,23]. These findings suggest just how beneficial animal companionship can be during times of personal struggle. Though many people experienced significant stress, financial concerns, and decreased quality of life due to the pandemic, pets maintained their positions as helpful sources of support during these times [17,22]. In fact, results from a study of a Spanish population during the lockdown found that the more severely a person’s quality of life was impacted, the more that person was likely to depend on their pet during isolation [17]. There are many potential explanations for the ways in which pets have been found to be beneficial to human mental wellness during the COVID-19 pandemic. Long recognized as a means for providing essential social support [6], pets were found to assist their owners in coping with the dramatic decrease in social interaction that came with the pandemic [17].

Sharing a home with a pet could also provide protective measures in terms of inspiring a person to continue with existing routines as normally as possible. For example, people who share their home with a dog might be required to adhere to breaks for urination and defecation, feeding, and exercise schedules [18], providing a sense of normalcy even on the most non-routine days of pandemic-related lockdown. In some countries, the need for dog walking was recognized as an essential outing, and dog owners were permitted to leave their homes to exercise their canine companions [17]. In addition to helping owners adhere to routines, pets also helped their owners reduce pandemic-related stress and anxiety. According to Volume 4 of the APPA Pulse Survey, as many as 73% of pet owners stated that spending time with their pet helped reduce stress and improve wellbeing during the pandemic [20]. A survey of 4105 dog owners found that over half of respondents reported that their dog was helping reduce feelings of distress [24]. In the UK, 86% of pet owners said that their pet was helping them cope emotionally during social isolation [25]. Many pet owners have reported that their pet helped them maintain a regular schedule, cope with uncertainty, be compassionate towards themselves, and find purpose in their lives. Specifically, 75% of people said they were turning to their pets three or more times per day to feel better, with 16% turning to their pet 10 or more times per day [26]. Pets helped their owners stay physically active. Many pet owners were spending more time outside with their dogs and even exercising more with their pets in general mid-pandemic [26]. Forty-two percent of dog owners reported walking their dog more than before the pandemic [27]. A separate study suggested that dog owners working from home during the pandemic were more likely to socialize with others, achieve a healthy amount of activity, and take at least one 15-minute walk during the workday than people without dogs [28].

Those who shared their home with animals were not only more likely to benefit from the HAB during the pandemic, but were also inclined to devalue the cost of pet ownership in the wake of COVID-19. More than just financial considerations are at play when considering the cost of having an animal. When a person is responsible for a pet, they tend to have less freedom of choice in terms of their ability to leave the home for longer periods of time [17]. Given the fact that freedom of choice for activities outside of the home was limited by the pandemic, the perceived burden of pet ownership as reducing one’s ability to leave the home as one pleased was decreased [17].

## 3. The Influence of COVID-19 on Accessing HAI

Prior to the COVID-19 pandemic, there were a variety of ways a person who did not share a home with an animal could still benefit from interacting with animals. Even without having a pet, we could interact with other peoples’ companion animals in public locations such as parks and outdoor dining areas. Places like zoos, aquariums, and sanctuaries were open to the public so that people could access HAI with a wide variety of species. Countless people all over the world incorporate their love for animals into their volunteerism, helping at local animal shelters, or engaging in animal-assisted activities (AAA) [29,30]. However, when the pandemic required us to significantly alter our ways of living and turn to spending much more time isolated in our homes, many of these interactions were suddenly halted. By first recognizing the grave impact the pandemic had on all types of animal engagement, we can appreciate how we came to a place of improved recognition for the power of the human–animal bond in the midst of these challenging times.

As we collectively began to consider how the pandemic might impact our pets, we also shifted our behaviors to best accommodate HAI and allow it to continue during these times. Changes were noted in the ways we interact with and care for pets in the home, and there were significant shifts both in the service animal and therapy animal industries, such as how service animals were trained and how AAA was administered, that will be explained further in this paper. These shifts speak to the flexible and resilient nature of the HAB, suggesting innovative means for expanding access to the HAB through HAI even when society begins to normalize as the pandemic becomes more controlled.

### 3.1. Pandemic Isolation Measures Overview

Although the pandemic has impacted the entire globe, each country has responded to this global health crisis in different ways. Infection rates and subsequent hospitalizations spiked early on for some countries, such as Italy, calling for extensive social distancing guidelines that have persisted throughout the course of the pandemic [31]. Countries such as Uganda went into action before ever having a confirmed case, limiting travel, mandating quarantine for incoming visitors, and preventing large gatherings of people [32]. Even as the vaccine became available, researchers in the U.S. noted differences in individuals’ acceptance and willingness to receive the vaccine [33]. Across the varied ways in which countries, governments, communities, and individuals responded to COVID-19, lifestyles have certainly shifted. People have had to cope with social isolation, workplace differences and/or lack of employment, new norms such as masking and wearing gloves, and even shifts in experiences of depression and anxiety [34,35]. As human behavior has changed given the protective measures that have come into place as we respond to the pandemic, the pets with whom we share our lives have also been impacted [36].

### 3.2. Impact on Pet Spending & Ownership in the Home (Impact on Human Behavior in Pet Ownership)

COVID-19 has had various impacts on pet ownership. Survey research conducted during the pandemic has resulted in reports of increased pet ownership. The American Pet Products Association (APPA), the leading trade association in the pet industry made up of 1000 pet product manufacturers, their representatives, importers and livestock suppliers, commissioned four volumes of the COVID-19 Pulse Study of Pet Ownership During the Pandemic, an online survey of pet owners and non-owners in 2020. The purpose of this market survey research was to collect data and insights into pet ownership as well as pet product purchasing and service consumption during the pandemic. Since 1988, APPA has published the APPA National Pet Owner’s Survey, the industry’s most comprehensive consumer research study of demographics, buying habits, and other traits of owners of dogs, cats, fish, birds, reptiles, small animals, and equines.

APPA conducted their COVID-19 Pulse Survey in four volumes or waves. The first wave (Volume 1) of the COVID-19 Pulse Study of Pet Ownership During the Pandemic survey was conducted over one week in May 2020 among a sample of 2006 nationally representative individuals. The second wave (Volume 2) was conducted using the same methodology over one week in June 2020 and had 2008 respondents. The third and fourth waves (Volumes 3 & 4) were conducted over one week in September 2020 and one week in late November/early December 2020, respectively. Both the third and fourth waves each had 2007 respondents. The questionnaire surveyed both pet owners and non-pet owners. While figures vary, APPA reported that approximately 10% of their survey respondents got a new pet during the pandemic as of December 2020. Extrapolating this figure, an estimated 11.38 million U.S. households acquired a new pet during the pandemic. By generation, Gen Z or respondents aged 18–23 (18%) and Millennials or respondents aged 24–39 (15%) reported significantly higher percentages in new pet ownership resulting from the pandemic, as compared to Gen X or respondents aged 40–55 (10%) or Baby Boomers or respondents aged 56–74 (2%). In December 2020, the percentage of people who said they may have to give up their pet was the lowest of all the Pulse survey waves at just 8% [20]. Other surveys have reported even higher numbers of pet acquisition during the COVID-19 pandemic. The Mars Petcare BETTER CITIES FOR PETS Pets in a Pandemic Report found that one in three pet owners welcomed a new pet in 2020, with more than half (58%) doing so for companionship, and more than one-third (36%) reported getting a new pet to avoid or alleviate depression.

As a result of new pet ownership, pet spending has remained steady. According to the APPA Pulse Study Volumes 1, 2, 3, & 4, spending on pet food, supplies, and services has stayed consistent during the pandemic despite economic challenges and uncertainty. Between May and June (of 2020), no significant shift in the effect of the economy on pet spending was observed. When asked about pet spending habits in June 2020, 64% of pet owners spent the same amount of money on their pets as they did pre-pandemic. 21% had spent either a little more or a lot more on their pets as compared to their existing spending habits. Just 15% had either spent a little less or a lot less on their pets since the onset of the pandemic. The percentages of pet owners who shopped for pet supplies remained consistent during that same timeframe. While the percentage of respondents who reported being concerned about their finances over the coming year increased from 55% in September 2020 to 61% in December 2020, the APPA Pulse surveys found that this concern has not led to notable changes in pet product purchasing behavior, and that pet product purchasing has remained consistent across all four survey waves. Changes noted include a slight decline in in-person shopping for vitamins/supplements from 57% in May 2020 to 53% in December 2020, and an increase in pet owners choosing to shop online through a retailer and either have supplies delivered to them or picked up at the store (44% in May 2020 to 47% in December 2020). Further, more pet owners reported stocking up on food, treats, medication and vitamins/supplements for their pets in December 2020 than in each of the previous waves. For example, 57% of pet owners in December 2020 stated they stocked up on pet food on their most recent shopping occasion in contrast to 52% in October 2020, 53% in June 2020 and 55% in May 2020 [20].

It is important to recognize that the impact of COVID-19 was different for different pet owners. Among respondents of Volume 4 of the APPA Pulse Study, those with children were significantly more likely (68%) to be very concerned about their finances over the next year than those without children (57%). Additionally, those with incomes of less than $50,000 per year (66%) were more likely to be very concerned about finances over the next year than those making over $100,000 annually (52%). Millennials reported the highest percentage of those financially impacted by COVID-19 (59%); a level that rose steadily since May 2020 (48%). Throughout the Pulse Studies, general levels of concern about the expense of pet ownership during the pandemic had never risen above 30% among any generation over the course of the study [20].

While some budgets may have tightened during the pandemic, time dedicated to pet ownership seemed to increase. Pet owners have largely reported spending more time with pets—a direct result of spending more time at home—and that this extra time together has improved their mental health and wellbeing [24]. For animals with chronic medical issues, nursing care was easier to provide during the pandemic because owners were home full time to tend to an animal’s needs. Some pet owners are already thinking about how they will adjust to the new status quo with their pets. Data has shown this additional time spent with pets has indeed resulted in greater attention towards pet care. Pet owners reported being more attuned to their pets’ health and wellbeing than they were before the pandemic. According to results from a Banfield Pet Hospital survey, 9.2% more juvenile dogs and 12.4% more juvenile cats were brought into Banfield for veterinary visits in 2020 compared to 2019, the first increase in the percentage of juvenile pets seen at the practice in 10 years [37]. The survey also found that pet owners were more committed than ever to getting their pet to the vet. Visits increased 20.4% for kittens and 14.3% for puppies in 2020. This indicates that, despite the barriers to accessing veterinary care during the pandemic, the veterinary industry has persevered.

The difference between essential and nonessential workers may have played a large role in the individuals’ ability to care for and spend time with a pet during the pandemic. In addition, there may be disparity in the socioeconomic status (SES) of these pet owners, with the ability to care for a pet relating to a person’s ability to work from home and/or maintain an affluent lifestyle. Individuals of lower SES are less likely to have employment security or have the ability to work from home [38]. It is possible that pet keeping may not have been a priority for individuals of lower SES, whereas individuals of higher SES were more likely to keep their jobs and work from home [39], and thus maintain the privilege of keeping and caring for their pets. Although there is disparity in the practice of pet ownership between classes, it is clear that caring for pets has remained an important concern and source of stress throughout the pandemic regardless of SES [40].

#### Effect of COVID on the Pets in the Home 

Whenever our human behaviors change enough to alter lifestyle, there is a chance the animals with whom we share our lives with will also be impacted [41]. Aware of this possibility, many pet owners had concerns about the implications of COVID-19 and its aftermath on their animals. In a 2020 study [17], roughly 62% of dog-owning participants worried about their ability to go on walks while social isolation was necessary, while cat owners tended to be more concerned with not being able to access the vet and/or medications their cat needed [42]. Interestingly, a separate study suggested that dog owners working from home during the pandemic were more likely to socialize with others, achieve a healthy amount of activity, and take at least one 15-minute walk during the workday than people without dogs [28]. Some pets may have benefitted from the pandemic, from increased adoptions and fosters, spending on pet products, and time spent with the pet [28]. However, we cannot ignore that there may have been more adverse consequences as well. The increase in adoptions and fosters with the goal of getting animals out of shelters and into homes was an obvious benefit. These animals entered a life where owners were home at all times and were dedicated to them. However, there may have been some conditions in which new adoptions or fosters were not best suited for the animal. First, those who obtained a new pet during the pandemic with no prior pet ownership experience may not realize the true, long-term needs of the animal. Second, newly acquired pets change the social dynamic for both people and other animals in the household. If not properly introduced, animals can create tension or changes in the animal hierarchy [43]. Additionally, pandemic-related increases in the number of people in the household for extended periods of time could have created more tension and stress, making it more difficult for these new animals to adapt to their new homes. Finally, these animals may not have received proper socialization and training due to the social distancing mandates and lack of access to sources of socialization such as dog parks and day care, increasing the likelihood of behavior problems in the future and a potential decrease in welfare.

For the animals attached and highly bonded to their owners, they received comfort from the perpetual one-on-one time they shared with one another [24]. This personal and relationship-oriented time became part of a daily, regular routine for those able to live and work from home. Because they were observed more intently throughout the day with a watchful eye, problems that warrant veterinary or behaviorist attention were recognized sooner. They were also safer from the elements and accidents that may have resulted from taking them in public spaces such as dog parks.

However, we cannot ignore the consequences of the extended time spent with these animals that did not occur previously. It may have been frustrating for some animals to have their owners at home and not give them constant attention. Pre-pandemic, owners may have spent a significant amount of quality time with their pet and paid all their attention to the animal while physically in the home, such as on the weekends or in mornings or evenings. Work and home lifestyles were maintained as separate with clear boundaries for the animal regarding what to expect while the owner was at home. The pandemic altered this dynamic almost instantaneously for those able to work from home. Suddenly, the pet owner was home all the time, yet remaining at a desk and paying attention to the computer rather than the pet. Although pet owners indicated that pets created welcome distractions during the workday [28], the pet may have been frustrated with the owner due to not receiving enough attention, resulting in attention-seeking behaviors that were never satiated or quelled.

In a 2020 study that investigated the effect of the lockdown on homes with pets in Spain, dog owners reported that though new behavioral problems in dogs did not commonly develop, existing issues such as vocalization, fear of noises, attention-seeking behavior, and nervousness increased for some dogs [17]. Cats, which can often be more sensitive to human contact [44], were also reported to show some increases in attention-seeking behavior over the course of isolation with their owners [17]. Any behavioral changes that our animals have displayed throughout the pandemic are worth noting, as they could become problematic when the time comes for humans to leave the home more frequently and for longer durations of time [17].

Furthermore, due to a more sedentary lifestyle, many pets may have gained weight. Pets who had dog walkers or who were brought to pet daycare normally had opportunities to release energy, but these outlets became suddenly unavailable due to the pandemic. For those owners who preferred not to or who were unable to exercise their dogs without the support of these services, more problems and issues with welfare could have resulted.

How the animal responded to the pandemic is likely a reflection of how the human responded to the pandemic. Throughout this time of isolation, many pet owners have reported they have noticed behavioral changes in their animals. For many humans, the impact of confinement has led to decreased perceptions of quality of life [17]. These negative feelings from owners may have spillover effects on animals’ quality of life as well. Though pets have often helped to alleviate humans’ negative feelings associated with confinement [17], the toll on some animals has led to new or worsening behaviors on behalf of the pet. While some pets were adversely affected, others either were not impacted at all, or benefitted from the changes associated with the pandemic. Each animal may have a different response depending on the individual. When humans experience increased anxiety and depression, animals have the ability to read those feelings and potentially act as an empath for the human [45]. A person might become more volatile, and in turn, create feelings of hostility or uneasiness in the animal. A person who adjusted easily to the pandemic may have an animal that adjusted similarly.

### 3.3. Impact on Service Animal Industry

Defined by Title II and Title III of the Americans with Disabilities Act (ADA), a service animal is defined as “any dog that is individually trained to do work to perform tasks for the benefit of an individual with a disability, including a sensory, psychiatric, intellectual or other mental disability”. Also referred to as “assistance animals”, service animals are permitted to accompany their handler into public locations. Any business or facility that serves the public generally must allow service animals to accompany people with disabilities in all areas of the facility where the public is allowed to go. A service animal can assist their handler in a variety of ways, both in terms of the specific task they have been trained to help their handler with and by aiding their handler in secondary benefits such as social engagement [7]. Given the significant role these animals play in the lives of their human counterparts, these working dyads experienced unique challenges due to the COVID-19 pandemic. To adequately capture these experiences, a few service animal industry representatives contributed to this paper via personal correspondence with the authors.

Aside from their relationship with a service animal, people with disabilities faced specific challenges that impacted all areas of their life during these times. People with visual impairments often depend on touch, a sense that has been made less available due to infection prevention guidelines (L. Kenney, personal communication, 11 May 2021). Masks have also posed a unique challenge for many people with disabilities, as lip reading is prevented, and voices often are hampered by face covering. Consequently, service animal handlers experienced unique challenges associated with COVID-19 that set a foundation for struggle when working alongside their animals (L. Kenney, personal communication, 11 May 2021).

Service animal handlers were gravely impacted at the individual level. The training that active service animals had previously been through could not have predicted the new norms that were born of the COVID-19 pandemic. Animals trained for handlers with sensory impairments were not familiar with the need to be socially distant from others in public settings. With this in mind, some service dog handlers reported having experienced conflict with other people when they were led by their dogs into close physical proximity to others (L. Kenney, personal communication, 11 May 2021). Forced into social isolation, both the humans and the animals who make up these pairs were reported to experience hardships related to the inability to socialize (C. Youell, personal communication, 18 May 2021).

In addition, service dog organizations rely heavily on volunteer support to carry out their missions effectively. While some of the service dog organizations reported even higher levels of volunteer engagement during the course of the pandemic, connecting with these volunteers required innovation and ingenuity, as many of the traditional means of volunteer engagement were not possible during these times (L. Kenney, personal communication, 11 May 2021). Along with operational changes, these organizations experienced hardships in their ability to fundraise, provide education and training, and connect with communities through their typical avenues (H. Wilkerson, personal communication, 26 April 2021). Limited by these restrictions, some service animal organizations were unfortunately required to lay off staff and curtail their services due to COVID-19 (L. Kenney, personal communication, 11 May 2021).

Having realized the vast impact the pandemic had on individual service animal dyads and on the industry as a whole, leaders from service animal organizations quickly pivoted to find new ways to meet their mission in these challenging new times. Once it was safe to do so, these organizations continued with their training courses in innovative ways, offering smaller class sizes cognizant of social distancing guidelines (L. Kenney, personal communication, 11 May 2021).

“Challenge and opportunity are two sides of the same coin,” reported one service animal professional (L. Kenney, personal communication, 11 May 2021). Though the times were hard, they inspired creativity and innovation that has influenced both program development and training techniques that can be used with service animals. Service dog organizations now depend on more technology than ever before. Platforms have been created so these organizations can connect with clients remotely and offer training to the animals in virtual meeting spaces (L. Kenney, personal communication, 11 May 2021). There has been a transition from in-person to virtual events for many of their educational offerings, resulting in the ability to reach and raise awareness for a larger audience than pre-pandemic times (H. Wilkerson, personal communication, 26 April 2021). Even fundraising efforts have transitioned to online platforms, allowing the organizations to continue meeting their essential missions to match people with service animals (L. Kenney, personal communication, 11 May 2021).

#### Effect of COVID-19 on the Service Animals Themselves

Similar to pet owners, service animal organizations and handlers share a significant concern that some service animals might exhibit behavioral changes due to the lack of stimulation and interaction resulting from this period of time (L. Kenney, personal communication, 11 May 2021). As with other observations relating to pets, service animals faced health concerns as a result of COVID-19, with some animals experiencing weight gain and other animals experiencing a difficult time accessing regular preventive veterinary care, likely due to pandemic-related restrictions. The handlers of these animals also faced challenges at the individual level. Access to training courses and applied practice was limited, and some clients expressed feeling less confident about re-enter society with their service animal when the time came (L. Kenney, personal communication, 11 May 2021).

The administrative aspects of service animal training were also seriously impacted by the pandemic. The general operating procedures of these organizations were challenged, requiring staff changes and often expensive adaptations to providing client services and animal training in virtual capacities (L. Kenney, personal communication, 11 May 2021). Programming stalled completely for many of these agencies for a period of time, creating a backlog of candidates seeking to be paired with service animals (C. Youell, personal communication, 18 May 2021). Dogs that were in training at the time of the pandemic’s emergence spent more time than normal in their foster homes, and the breeding of new service animals was curtailed (L. Kenney, personal communication, 11 May 2021). Even the organizations that utilize rescued animals for service dog training experienced shortages in new canine candidates, likely due to the sudden increase in shelter animal adoption by the general public (H. Wilkerson, personal communication, 26 April 2021). For the dogs that were already in these programs at the start of the pandemic, there was concern that the training process for these animals would be hindered by the lack of socialization necessitated by social distancing measures (L. Kenney, personal communication, 11 May 2021).

While many people reported they had more time to take exceptional care of their pets during the pandemic, others did not. The handlers with disabilities who typically sought care outside of the home for needs such as grooming and nail trims likely suffered a hiatus [46]. In fact, because handlers may have been unable to access this care during the pandemic, they may have been compelled to take on these tasks themselves. This could have resulted in injuries for both owner and animals when owners were unable to treat or handle animals properly.

### 3.4. Impact of COVID-19 on the Therapy Animal Industry

A therapy animal is an animal that has been evaluated based on their ability to safely engage in partnership with their human handler across a wide range of settings [47]. These animals provide physical, psychological, and emotional benefits to the people they interact with, and they can be called into various activities and/or professional treatment plans to inspire a sense of wellness for those interacting with them [47]. There is a growing body of research to support therapy animal interventions, with findings that point to the intervention’s ability to bolster participant outcomes for people of all ages and all walks of life [12,14].

Perhaps one of the most challenging barriers faced by the therapy animal community when the pandemic first began related to the fact that therapy animals are often called upon to bring wellness to populations that are most vulnerable to the COVID-19 virus. Therapy animal programming within medical facilities, residential care homes, and schools all suddenly came to a stop. Professionals who normally incorporated therapy animals into their treatment plans were challenged in their ability to deliver this important therapeutic intervention. Although disruptive, this change was not only essential to protect the people who receive therapy animal visits, but was also an essential consideration in protecting therapy animals and their handlers as we waited to learn more about the COVID-19 virus. Though the change was necessary, people all over the world experienced the negative impact associated with ceasing therapy animal interventions.

Organizational data from Pet Partners, one of the world’s leading therapy animal organizations, speaks to the grave impact of COVID-19 on AAI. Whereas roughly 33,730 therapy animal visits were logged in 2019, only 9176 visits were logged in 2020. Prospective handlers were also largely unable to join the organization during the pandemic, as all new teams were required to attend an in-person evaluation to register a therapy animal. During the 2020 year, an average of only four evaluation events were scheduled each week across the globe, as compared to the 26 evaluation events per week that typically took place in 2019. This dramatic shift in evaluation availability led to a greater than 50% decrease in new therapy animal teams in 2020 as compared to 2019. Even as time has progressed and vaccines have started to become available, many handlers report a lingering uncertainty about how comfortable they are with moving forward with therapy animal evaluation in 2021. When Pet Partners polled their volunteer teams who were set to renew their registration in May of 2021, over 50% of the survey respondents indicated that they were not sure if they would be comfortable attending an evaluation with their animal in the first half of 2021.

Almost immediately after the need for social isolation was realized, therapy animal teams all around the world went to work to find creative solutions for maintaining contact with the populations that had come to depend on interactions with therapy animals as a crucial means of support. Therapy animal teams who had been visiting within a given facility for years contacted facility staff to set up virtual opportunities for engagement with residents who desperately needed connection. People from all walks of life were able to check in with handlers and their therapy animals on video platforms, sometimes for a casual conversation and other times doing more formalized activities, such as working through obedience cues or practicing reading skills with the animal on the other end of the screen. Window visits became popular, allowing for a person to visit a facility through the safety of a window while incorporating innovative strategies for encouraging the therapy animal to interact in this new capacity.

Even when virtual and window visits were not possible, therapy animal teams were determined to find other ways to continue to connect within their populations of interest. Some teams began to write letters or share animal-focused crafts with facilities in their communities to encourage the sentiment of the love of animals. These kinds of activities suddenly became both massively popular and widely utilized to combat feelings of loneliness within populations who were most isolated by the pandemic. Pet Partners created a social media group page in April of 2020 to serve as a place for teams to upload content to be shared with facilities all around the world. In just eight months, over 2000 members joined the group, ultimately reaching a network of hundreds of facilities who use the page’s content to engage with the patients, clients, and students whom they serve.

#### 3.4.1. Animal Related Engagement

Realizing the auspiciousness of these creative new activities, Pet Partners’ staff worked to determine whether there was any empirical basis to support the efficacy of these initiatives. A literature review on animal-related stimuli pointed to various ways in which activities with either passive involvement of animals or activities with model animals have proven to be beneficial for certain populations [13,48,49,50]. This is a construct that had been frequently considered in research on populations with dementia, finding that activities such as watching puppy videos, interacting with plush and robotic animals, and even coloring pictures of animals positively impacted variables including affect and engagement while decreasing agitation levels [13,48,49]. Installing a fish aquarium in a residential care facility for people with dementia even led to improvements in patient behavioral measures, as well as increases in staff job satisfaction for caretakers [51]. Promising findings of this nature were not limited to populations with memory impairment. Viewing cute animal videos within a more generalized population has been correlated with multiple positive outcomes. Across various studies, animal video viewership is associated with positive affect, increased resourcefulness and productivity, better moods, and even improvements within interpersonal relationships [50,51,52,53,54].

Though research on this topic is foundational, it provided empirical support to explain the anecdotal experiences of therapy animal teams who were finding tremendous success connecting over the love of animals in new ways due to the pandemic. To operationalize and support these efforts, Pet Partners coined the term Animal Related Engagement (ARE), which describes any engagement opportunity that allows participants the benefits of the human–animal bond by encouraging the remembrance of feelings that are commonly associated with interaction with a live animal [47]. Although this initiative was motivated by challenging times related to the pandemic, it is one that is expected to continue long into the future. ARE can be employed in any instance in which a therapy animal team is either not available or not appropriate for a given population. It is a tool that might be used to prepare a facility for upcoming AAI, or it may be utilized when AAI services must suddenly be terminated. To continue to support this great work and make HAI as widely available as possible, a collection of free resources including virtual visit guides, a virtual reading program, and a therapy animal coloring book are freely available on the Pet Partners website.

#### 3.4.2. Effect of COVID on Therapy Animals Themselves

Therapy animals that typically remained at home and only occasionally travelled to engage in therapy work may not have experienced a great deal of change on a day-to-day basis if they stopped therapy work completely and remained at home full time during the pandemic. Therefore, COVID impacted them as pets in the household, as discussed above. However, it is possible animals that were accustomed to, and benefitted from, regular socialization through therapy visits may have been frustrated by the sudden onset of isolation. Engaging in therapy animal programming is an important bonding activity for many therapy animals and their handlers, and this shared role was challenged during the pandemic. In contrast, perhaps the long reprieve from visits was good for therapy animals that needed a break or were ready to retire from their role.

Therapy animals that engaged in ARE may have a different experience working with their handlers virtually from home. They may not appreciate or understand why they are being restricted to the confines of a computer or phone frame for extended periods of time. Some animals could become fearful of owners following them with phones, computers, or cameras; specific training to prepare these animals was necessary to protect their wellbeing if they were involved in this kind of new programing. On the other hand, ARE also creates intentional engagement and activity for the owner/handler, which may positively affect their quality of life and strengthen the human–animal bond between the therapy animal and owner/handler. Additionally, the animals were ultimately safer from the physical and infectious disease risks that therapy animal work may present.

## 4. Re-Emerging into Society Together

The world has significantly changed due to the impact of the COVID-19 pandemic. As vaccines become more readily available and the population prepares to safely reintegrate into society, we must not forget that, just as the shutdown impacted our animals, so will re-entry. Everyone’s life, whether human or non-human animal, has changed. Many have had over a year to adapt to these new lifestyles of spending more time at home and in social isolation. Whether an animal’s behavior has changed for better or worse because of the lifestyle changes of their human counterparts [41], we must do all that we can to make future transitions as comfortable as possible for our animals. Whether we share our lives with a pet, service animal, or therapy animal, we are obliged to make a commitment to protect and advocate for the welfare of that animal. We must be aware of the ways animals perceive the shift in norms due to the pandemic, and how to best prepare for responding to animals’ ongoing individual needs.

As pandemic-related restrictions ease, there will be new norms that both humans and animals must adjust to. Motivated by our renewed appreciation for the HAB, now is the time to recommit to our positions as advocates for animal welfare who are prepared to respond to the world in a way that sets our animals up for success.

### 4.1. Acclimating Pets in the Home to the New Status Quo

As pet owners return to normal life post-pandemic, they may encounter challenges when they no longer spend their time exclusively at home with their beloved pets. A survey of dog owners in the United Kingdom revealed an increase in new behavior problems as a result of the pandemic and reported that owners were concerned about their dogs’ ability to adapt post-pandemic [36]. Young pets acquired during the pandemic are unlikely to know a life outside of the pandemic. They are accustomed to an owner who is always at home and may be content to remain at home with them. This will mean a dramatic change when owners return to work and the animal is left alone for extended periods of time. The animal may not have received the proper training and socialization with people or animals since resources such as group training classes and puppy daycare were halted. Similarly, even pets owned prior to the pandemic may be subject to socialization challenges with unfamiliar people and animals. Some pets may be more reactive to strangers due to lack of exposure. Their response to strangers may need to be gauged and mediated, preferably with positive reinforcement techniques [55]. Trainers, behaviorists, and veterinarians may need to be integrated for animals having a difficult time coping with the exposure to these conditions.

When prompted, pet owners have indicated they intend to continue to take better care of their pets’ health going forward and reward their pet with better quality care in return for their animals’ being there for them during the pandemic [56]. This is important, as pet owners should be aware of their pet’s health and safety needs prior to their reintegration to public spaces, such as daycare and dog parks. For example, pets may not have received the necessary vaccinations due during the pandemic, for various reasons including limited access to veterinary care and decreased risk of exposure to infectious disease with a more inactive lifestyle. Therefore, they should receive the proper preventive care as they return to a more active lifestyle post-pandemic. Counseling with a veterinarian is key to providing the best care of pets. The availability of veterinary telehealth has increased and may continue to remain some owners’ preferred method of receiving veterinary care [57]. Additionally, online pharmacies and pharmacies that deliver may continue to thrive after COVID-19.

While more than half (56%) of pet owners have reported they plan to lessen their social activities and try to be home as often as possible to mitigate the sudden change for their pet [56], not everyone has the ability to do so. For those owners who are unable to remain at home, arrangements should be in place to provide the same or a similar level of care. A major concern for people spending extended periods of time at home with their pets is the potential provocation of separation anxiety once they return to work [36]. Pets that became accustomed to having someone always in their presence are now prone to experience separation anxiety once their owners begin to spend less time at home. In contrast, the pets that had separation anxiety issues prior to the pandemic and required behavioral medications may have improved significantly with owners being home to regulate and modify [58]. While these animals may have relied less on medications during the pandemic, it is possible that upon their owners’ return to normal work and separation, there may be a strong need for dependence on medications once again.

For those pets with separation anxiety and adverse behavior problems when the owner is not at home, it is critical the transition be made gradually to give the animal an opportunity to adjust to change [59]. If possible, it would be ideal for a human being to remain home with the pet. Realistically, since this is not always possible, the owner should leave for short periods of time at first, coupled with positive reinforcement and the avoidance of excitement upon the owner’s return home. The response of the animal should be monitored to see how they cope with the transition. In addition, leaving items that smell like the owner may provide the pet with the comfort that something of the owner remains. Finally, technology may assist in the form of live video surveillance of pets at home. Many advanced cameras have capabilities for two-way microphones so that the owner can still observe and talk to the pet virtually. Some treat dispensers have video, sound, and the capability of dispensing treats remotely. These are all potential ways for owners to remain engaged with pets from afar to mitigate separation anxiety and help the owner cope with missing their beloved pet.

### 4.2. Acclimating Service Animals to the New Status Quo

Proper adaptations for service animals include the considerations previously discussed for pets in the homes. However, what makes service animals particularly different is their need to return to public spaces. Most of these service animals have been out of work and practice during the pandemic. Therefore, handlers should undergo education and training that prioritize the health and welfare of the dog in preparation for the return to working life. In many instances a dog may need to be retrained, as recall may be slow [60]. Animals may react differently to environments, travel, and circumstances they were familiar with before. Therefore, a slow and gradual return to work is advised.

Additionally, people should not expect their animals to return to full capacity or to perform as they did previously. A year or longer is a significant amount of time for disease to advance, with aging and musculoskeletal issues, such as osteoarthritis, prone to recur. Prior to returning to service, animals should visit with a veterinarian to evaluate them for any health concerns, fitness, condition, and preventive health. In particular, nutritional counseling may be warranted. For service dogs that were sedentary during the pandemic, weight gain due to inactivity may be a significant issue. Therefore, reduction in caloric intake and increase in activity may be advised for those impacted.

### 4.3. Acclimating Therapy Animals to the New Status Quo

Many of the same considerations for service animals apply to therapy animals. The unique experience for therapy animals, as opposed to pets in the home or service animals, is the fact that therapy animals are asked to enter intimate zones of contact with people other than the handler/owner themself. This social engagement aspect adds a layer of risk for transmission of COVID-19 during therapy animal work. What the pandemic has fostered is the desire and need to be educated about zoonotic diseases in general. Instead of fearing AAIs because of this risk, properly educating handlers about the issue helps mitigate fears and is vital for a safe and productive return to the workplace. While the current body of knowledge of COVID-19 in domestic pets is sparse and ongoing, there is currently no evidence to show that animals are a significant source of COVID-19 transmission. Therefore, AAI organizations and facilities should re-emphasize standard operating and screening protocols for visitations, while maintaining a healthy regard for the conditions of zoonotic transmission.

It is also extremely important to reassure people that there are appropriate ways to protect against disease, including handwashing and patient selection. Handwashing is critical and mandatory practice before and after interacting with new patients. Additionally, fomites (inanimate objects that may be contaminated and have the potential to transmit infectious disease) such as leashes, bandanas, and collars should be washed regularly to minimize contamination. Some handlers with good intentions and/or miseducation may even attempt to vanquish COVID-19 by rubbing hand sanitizer into the paws or fur. However, these techniques are not deemed safe or effective, and could harm the animal. In addition, it is important to note that while it may cause fear and anxiety, a cough or sneeze from a person or animal does not necessarily indicate COVID-19 infection. While tests for COVID-19 in dogs and cats are commercially available, they should not be utilized as a first line diagnostic tool, as other diseases causing upper respiratory signs are more likely, and COVID-19 in animals is uncommon.

Handlers should also be educated on how to safely introduce themselves to participants, and should only visit with those who wish to have a visit from an animal. For example, it may be prudent for handlers to disclose their vaccination status and that they are healthy and asymptomatic. The handler may also choose to visit only with individuals disclosing the same criteria of being vaccinated, healthy, and non-symptomatic. The specific AAI organizations may dictate their own guidelines in line with their local public health regulations, and it is important the information is clearly discussed with handlers for a safe return.

Because the world will be different after the pandemic, behavior should be reassessed in animals prior to returning to work as a therapy animal. The therapy animal should be deemed reliable, controllable, and predictable. In addition, the therapy animal’s reaction to unfamiliar people in masks, as well as to common scents, such as hand sanitizers and disinfectants, should be assessed.

We are also aware many people may not return to therapy animal work because their animals are no longer suited to the work, or because they may not want to take the health risk. It is important to educate these individuals about the true risks of COVID-19 transmission. If they still decide to no longer be an active therapy animal handler, they should be offered non-handling opportunities to remain engaged. This can take the form of assisting with tasks such as fundraising, communications, outreach, and education.

## 5. Suggestions for Future Research & Best Practices

While we can deliberate on the global effects of the pandemic on human–animal interactions, it is necessary to document this with accurate statistics and research. There are many HAI-related questions that can be asked of this pandemic. Formal studies surveying the landscape of HAI before, during, and after the pandemic are key to influencing change and the directions we need to go. One fundamental question that can be asked regarding this period of social isolation and loneliness is whether pet ownership truly impacted mental health. Were pet owners more resilient than non-pet owners during COVID-19? From the animal perspective, it would be valuable to know the true incidence of behavior-related disorders and their association with the pandemic and social isolation. It would also be interesting to capture the behavior, temperament, and personalities in addition to the long-term health of animals adopted during the pandemic. Post-pandemic, we will benefit from having information regarding adoption and relinquishment rates in conjunction with the pandemic, and any factors, such as formal education, outreach, and training, that enhanced the adoption and retention of these animals. Moving forward, there is also a need to understand further the effects of COVID-19 on animal health and transmission, so we can educate the public about these issues. A lack of evidence for specific risk factors for increased transmission of COVID-19 in domestic animals remains.

There is still much to know about COVID-19 and pets, and specifically, how to re-introduce therapy animal teams in the workspace while keeping pets and participants safe and healthy. For therapy animals and service dogs, it will be revealing to note how many return to work, as well as the transition strategies that prove to be most favorable. In addition, it will be interesting to observe how many animals attempt to return to work but are unable to, therefore warranting retirement of the team. For those who are unable to work, or for those animals that may not have been appropriate for in-person AAA and AAT, they may still be able to participate safely in ARE. Therefore, it will be interesting to determine if ARE and virtual visits are comparable to in-person AAIs in efficacy regarding human outcomes and safety for human and animal welfare. Additionally, it will be informative to document which methods facilitate a smooth reintroduction of therapy animal teams into the workplace, and effective methods of education on COVID-19 and zoonotic diseases for handlers and participants.

For pets, the statistics of pet ownership, adoption rates, and relinquishment rates in the future cannot be accurately foreseen. Although initial data suggest relinquishment rates were not higher after COVID-19 lockdowns were lifted [18], there is still a real concern the reported increase in pet adoptions from shelters and rescues may result in increased pet relinquishment once the pandemic and related restrictions have subsided [25]. More accurate data and information regarding adoption and relinquishment will become evident in the near future. Returning to life outside of the house may cause people to realize they are unable to provide adequate care for their pet. Potential reasons for relinquishment as people return to a more normalized way of living include not having enough time to care for their pet, increased need/desire to travel, and behavioral changes in pets that result from their human companions’ new ways of being. To combat this, education and counseling for these pet owners through veterinarians, behaviorists, trainers and other professionals is necessary. For those who are no longer home to care for active dogs, increased access to daycare and dog walkers will be helpful. Even when all of these considerations are made, there may be some situations in which rehoming a pet truly is in the best interest of the pet’s welfare [25].

Ultimately, to prevent relinquishment and keep people and pets healthy and happy together, a societal change for people and pets is desirable. People have become accustomed to spending more time with their pets, and as a result, they are expressing an increased desire to maintain strong human–animal bonds. For pet owners, many may be fortunate enough to have employers who continue to allow them to work exclusively from home, or who perhaps allow more flexibility than prior to the pandemic [61]. In fact, 67% of pet owners who worked from home during the pandemic reported they would like the ability to take their pets to work [62]. Many pet owners have expressed concern over having to leave their pets behind when restrictions lift and life goes back to “normal”. It is important to realize that in bringing people back into the workplace, policies regarding companion animals may need to reflect these desires. Pet owners may be more demanding in regard to having pets in the workplace, especially given the benefits of pets in the workplace when facilitated properly. People find more efficiency and value in being able to work in the presence of pets. Previous research has demonstrated that working groups are more cooperative, comfortable, friendly, and attentive when a dog is present [63] and that pets make the work environment more comfortable and less stressful, and boost morale [64]. Research conducted by HABRI in partnership with Nationwide found that 90% of employees in pet-friendly workplaces felt highly connected to their company’s mission and fully engaged in their work, and were willing to recommend their employer to others. Not all workplaces will be suitable or safe for allowing pets to accompany their owners on a regular basis, if at all. Other pet-friendly policies, such as providing pet health insurance, allowing time off from work to care for a new or sick pet, and pet sitting services, are positive ways in which employers can demonstrate support for their pet-owning employees [65].

Even beyond work-life, many pet owners want to bring their pets with them wherever they go. Fifty percent of pet owners would be comfortable bringing their pets to outdoor stores, events, and patio seating at restaurants. Nearly two in three pet owners say they are likely to travel again in 2021, and about 60% want to bring their pets along when they do travel [62]. Similarly, rental housing regulations must change to accommodate more pet owners. Rental residents across the country indicate that pet-friendly housing is hard to find [66]. These strong sentiments will drive the demand for more pet-friendly places and spaces and changes in public access rights of pets. As we continue to recognize the growing significance of animals in our lives, there should be avenues to make it easier to live safe and healthy lives together with pets.

## 6. Conclusions

While the pandemic has underscored the importance of the human–animal bond as we have faced social isolation, loneliness, and unprecedented uncertainty, the pandemic has also changed life with animals, and is likely to continue to shape the ways HAI is accessed long into the future. We found that the new status quo has meant a new importance with regard to relationships with pets. As we have navigated these times together, the HAB has become stronger than ever as our animals have impacted us just as much as we have impacted them. We must recognize that, similarly to their effects on people, these changes have had unique influences on the animals with whom we share our lives in a variety of ways, a fact that has implications for HAI, pet care and welfare. Regardless of the new normal to which we have collectively become accustomed, we must recognize the call for change that will help us all to thrive in the future. Whether we share our lives with a pet, a service animal, or a therapy animal, we are called to make a commitment to advocate for that animal as a way of protecting their welfare and encouraging opportunities for them to adjust to the changing world as it normalizes post-pandemic. There is tremendous power available to us in this role as we become increasingly aware of the ways in which our animals perceive the world, and as we explore ways to best provide for their needs and strengthen the human–animal bond for our collective benefit.
